# Speech-based identification of suicide risk in patients with MDD: model development and 4-week follow-up validation

**DOI:** 10.3389/fpsyt.2026.1944154

**Published:** 2026-10-13

**Authors:** Yiang Liu, Nanxi Li, Qiushi Yang, Shuying Wang, Gang Wang, Lei Feng

**Affiliations:** Beijing Key Laboratory of Intelligent Drug Research and Development for Mental Disorders; National Clinical Research Center for Mental Disorders, National Center for Mental Disorders, Beijing Anding Hospital, Capital Medical University, Beijing, China

**Keywords:** follow-up validation, major depressive disorder, random forest, speech features, suicide risk, textual features

## Abstract

**Background:**

Patients with major depressive disorder (MDD) have a substantially higher risk of suicide than the general population, whereas current suicide risk identification mainly relies on clinical interviews and standardized rating scales, with limited objectivity and capacity for continuous monitoring. Speech is a low-burden and repeatable digital behavioral signal that can provide acoustic and linguistic information related to emotional state, cognitive processing, and verbal expression, and may offer an objective adjunct for suicide risk identification in patients with MDD. Accordingly, this study aimed to evaluate whether speech-derived features could support suicide risk identification in patients with MDD by comparing the discriminative performance of different speech representations, examining the added value of clinical symptom measures, and assessing model performance at a 4-week follow-up.

**Methods:**

Patients with MDD treated at Beijing Anding Hospital, Capital Medical University, with total scores of ≥14 on the 17-item Hamilton Depression Rating Scale (HAMD-17) were included. Suicide risk status was classified using a prespecified two-stage rule based on the HAMD-17 suicide item and the Beck Scale for Suicide Ideation–Chinese Version (BSI-CV). At baseline, clinical data and semi-structured interview speech were collected, and acoustic, traditional textual, and deep semantic features were extracted. Random forest models using different feature sources were developed and internally validated with fivefold stratified nested cross-validation. The textual-feature model and the model combining textual features with depressive symptom severity, represented by the Inventory of Depressive Symptomatology–Self-Report (IDS-SR) total score with the suicide-related item excluded, were further evaluated using 4-week follow-up data to determine whether their discriminative performance was maintained at a later assessment time point.

**Results:**

Among 248 patients with MDD, 92 were classified as high suicide risk and 156 as low suicide risk. In internal validation, the traditional textual-feature model achieved an area under the receiver operating characteristic curve (AUC) of 0.687, compared with AUCs of 0.604 and 0.641 for the acoustic- and deep semantic-feature models, respectively. The IDS-SR-only model achieved an AUC of 0.625. The models combining traditional textual features with the IDS-SR total score and with all clinical features achieved AUCs of 0.745 and 0.756, respectively. Among the 95 participants who completed the 4-week follow-up, 55 had a HAMD-17 total score of ≥14 at follow-up and were included in the 4-week follow-up validation analysis. In the 4-week follow-up validation set, the traditional textual-feature model achieved an AUC of 0.720, while the model combining traditional textual features with the IDS-SR total score achieved an AUC of 0.774.

**Conclusions:**

Speech-derived textual features showed potential value for supporting suicide risk identification in patients with MDD. Combining speech-derived textual information with clinical symptom measures may provide complementary information beyond standard symptom assessment. These findings suggest that speech-based approaches may support clinicians in identifying suicide risk among patients with MDD, although further validation in larger independent multicenter cohorts is needed before clinical implementation.

## 1 Introduction

Major depressive disorder (MDD) is a common and highly burdensome psychiatric disorder characterized by persistent depressed mood, loss of interest or pleasure, and a range of cognitive and somatic symptoms (1). Patients with MDD have a markedly elevated risk of suicide compared with the general population (2). High suicide risk in patients with MDD is not attributable to a single symptom or risk factor, but reflects the interplay of multiple clinical and psychosocial factors, including depression severity, hopelessness, negative self-evaluation, insufficient social support, and recent stressful life events (3, 4). Accordingly, suicide risk assessment in patients with MDD goes beyond determining whether suicidal ideation is present, it requires consideration of multiple dimensions, including the intensity of suicidal ideation, the specificity of suicide plans, previous suicidal behavior, acute risk factors, and protective factors (5). Moreover, suicide risk in patients with MDD may be difficult to detect and can fluctuate over time, further complicating the timely identification of high-risk individuals in clinical practice (6).

Although clinical interviews and standardized rating scales remain the main approaches for identifying suicide risk in patients with MDD, these methods still have limitations in objectivity, timely and reliable risk identification, and capacity for continuous monitoring. These limitations arise partly because clinical interviews and rating scales largely depend on patients’ active disclosure of suicidal ideation, suicide plans, and related behaviors, which may be influenced by current emotional state, therapeutic alliance, and the assessment context (7). In addition, clinical interviews and standardized rating scales typically require assessment by trained professionals, which may limit their feasibility for continuous monitoring in routine or real-world settings (8). These limitations underscore the need for objective, low-burden, and repeatable behavioral signals that can complement conventional suicide risk assessment.

Speech is one such promising digital behavioral signal. Speech production is not merely a vocal process, but involves respiratory control, vocal fold vibration, articulation, emotional regulation, cognitive processing, and language organization (9). As a result, speech can reflect mental state through both acoustic expression and linguistic content. At the acoustic level, features such as speech rate, pause patterns, fundamental frequency, loudness, and mel-frequency cepstral coefficients provide quantitative information about prosody, vocal intensity, spectral characteristics, and speech fluency, which may be influenced by emotional arousal and psychomotor activity (10). Beyond conventional handcrafted acoustic descriptors, self-supervised speech representations provide an alternative approach for deriving high-dimensional information directly from speech signals. These representations have also begun to be explored in mental health-related speech analysis. For example, Bauer et al. applied a pretrained wav2vec 2.0-based representation to automated MDD assessment using clinical interview recordings (11). However, their added value for suicide risk identification remains to be established. At the textual level, word choice, emotional expression, syntactic structure, and semantic content can more directly reflect cognitive processing, affective experience, and self-related appraisal (12). Based on these characteristics, speech features have increasingly been used for psychiatric disorder detection, symptom severity assessment, and monitoring of clinical changes (10). Methodological research in voice-based mental health assessment has also highlighted the importance of data quality and study design. Vilenchik et al. emphasized that factors such as class imbalance, label quality, sample characteristics, and recording conditions may influence the reliability and generalizability of voice-based depression detection, underscoring the importance of carefully designed data collection and validation procedures (13).

Previous studies have provided preliminary evidence that speech information can be used for suicide risk identification. Belouali et al. developed a suicide risk identification model based on speech collected during daily interviews with veterans and reported an AUC of 0.80 (14). Cui et al. developed a model based on adolescents’ self-evaluation speech, with an AUC of 0.92 (15). These studies suggest that speech signals may have potential for suicide risk identification across different populations and speech tasks.

However, applying speech-based approaches to suicide risk identification in patients with MDD remains challenging. Depression itself can induce acoustic and linguistic changes, including reduced speech rate, flattened prosody, increased pauses, reduced verbal fluency, greater negative emotional expression, and increased self-referential language (16). These depression-related acoustic and linguistic characteristics may overlap with speech features associated with suicide risk, thereby making suicide risk identification more difficult in patients with MDD. Several studies have examined speech-derived information for suicide risk identification in patients with MDD or bipolar disorders. Li et al. extracted linguistic features from transcripts of HAMD assessment recordings obtained from patients with MDD and healthy controls, reporting an AUC of 0.77 for the model based on suicide-related item transcripts (17). Min et al. developed an acoustic-feature-based suicide risk identification model in a sample comprising patients with MDD and patients with bipolar disorder, reporting modest performance with an AUC of approximately 0.62 (18). Lin et al. also examined a sample comprising patients with MDD and patients with bipolar disorder, and combined acoustic and word-frequency features from speech elicited by positive, neutral, and negative question-and-answer materials. The model based on speech elicited by negative materials achieved relatively better performance, with an AUC of 0.80 (19).

Despite these preliminary findings, several limitations remain in the existing evidence. First, previous studies often included healthy controls or patients with bipolar disorder, whereas studies specifically focused on suicide risk identification in patients with MDD remain limited. Second, previous studies have varied substantially in speech task design, feature extraction, and modeling procedures. Some studies used speech or textual content highly related to suicide risk assessment as model inputs, or performed feature selection and parameter optimization on the full dataset, which may increase the risk of data leakage and overoptimistic performance estimates. Third, most previous studies have relied on cross-sectional data for model development and internal validation, leaving limited evidence on how such models perform when applied to newly collected speech at later assessment time points.

Based on these gaps, the present study aimed to evaluate different speech-derived representations for suicide risk identification in a clinically homogeneous sample of patients with MDD and HAMD-17 total scores of ≥14. Acoustic, traditional textual, and deep semantic features were compared within a unified random forest framework using leakage-aware nested cross-validation. We further examined whether incorporating clinical symptom measures provided additional discriminative value beyond speech-derived features. Finally, the better-performing models were applied to newly collected speech at the 4-week follow-up to evaluate whether their discriminative performance was maintained at a later assessment time point. This design allowed us to compare multiple speech representations under a consistent evaluation framework while also examining their performance across assessment time points.

## 2 Methods

### 2.1 Study population

This observational clinical study recruited patients with MDD who received outpatient or inpatient treatment at Beijing Anding Hospital, Capital Medical University, between December 2024 and November 2025. The inclusion criteria were as follows: (1) age between 18 and 65 years, regardless of sex; (2) a current diagnosis of major depressive disorder according to the Diagnostic and Statistical Manual of Mental Disorders, Fifth Edition (DSM-5), without psychotic symptoms, confirmed using the Mini-International Neuropsychiatric Interview (MINI); (3) a 17-item Hamilton Depression Rating Scale (HAMD-17) total score of ≥14 at enrollment; (4) no modified electroconvulsive therapy within 14 days before enrollment; (5) the ability to understand the clinical scales and speech tasks used in the study. The exclusion criteria were as follows: (1) a diagnosis of other psychiatric disorders according to DSM-5, including intellectual disability, schizophrenia spectrum disorders, bipolar disorder, or mental disorders due to other medical conditions; (2) current severe physical illness that could affect speech production or completion of the speech tasks; and (3) a history of alcohol or psychoactive substance abuse or dependence within the past year.

This study was approved by the Ethics Committee of Beijing Anding Hospital, Capital Medical University (approval No. [2024] Scientific Research No. 386). All participants provided written informed consent before participating in the study.

### 2.2 Definition of suicide risk groups

Given the clinical need to identify patients with MDD at high suicide risk in a timely manner and facilitate early intervention, suicide-risk status was operationalized as a binary outcome using a study-defined two-stage, scale-based rule established before model development. This procedure combined clinician-rated information from the suicide item of the 17-item Hamilton Depression Rating Scale (HAMD-17) with self-reported information from the Beck Scale for Suicide Ideation-Chinese Version (BSI-CV). The HAMD-17 suicide item captures thoughts of death, suicidal ideation, and suicidal behavior based on clinical interview (20); however, as a single item, it provides limited differentiation of suicidal ideation severity, suicide planning, and related risk dimensions. The BSI-CV provides a more detailed assessment of the intensity of suicidal ideation, suicide planning, perceived self-control, and suicide-related preparatory behaviors (21). Accordingly, the two-stage procedure was intended to integrate complementary clinician-rated and self-reported information while reducing reliance on a single assessment source.

Specifically, all participants completed both the HAMD-17 suicide item and the BSI-CV. Under the prespecified two-stage classification rule, participants with a HAMD-17 suicide-item score of ≤1 were classified as having low suicide risk. Among those with a score of >1, a BSI-CV total score of >10 was used to classify high suicide risk; all remaining participants were classified as having low suicide risk. The HAMD-17 suicide item and BSI-CV were used solely to define the suicide-risk labels and were not included as predictors in the models.

### 2.3 Clinical assessments

At baseline, all participants provided sociodemographic and clinical information and completed clinical scale assessments. Participants who completed the 4-week follow-up were reassessed with clinical measures relevant to suicide risk grouping and model validation, including the HAMD-17, BSI-CV, Inventory of Depressive Symptomatology–Self-Report (IDS-SR), and Generalized Anxiety Disorder-7 (GAD-7). The Dimensional Anhedonia Rating Scale (DARS) and Clinically Useful Depression Outcome Scale with DSM-5 Mixed Features specifier (CUDOS-M) were administered only at baseline. All assessments were conducted by trained research staff following standardized procedures.

The HAMD-17 is a commonly used clinician-rated instrument for assessing the severity of depressive symptoms and has demonstrated good reliability and validity (20). It consists of 17 items, including nine items scored from 0 to 4 and eight items scored from 0 to 2, with a total score ranging from 0 to 52. Higher scores indicate more severe depressive symptoms.

The BSI-CV is a self-report instrument used to assess the severity of suicidal ideation (21). It evaluates attitudes toward life and death, intensity of suicidal ideation, suicide planning, and related suicidal tendencies. The scale consists of 19 items, each scored from 0 to 2, with a total score ranging from 0 to 38. Higher scores indicate more severe suicidal ideation and higher suicide risk.

The IDS-SR is a self-report instrument used to assess the severity of depressive symptoms and provides a comprehensive evaluation of depressive symptom domains (22). It consists of 30 items, each scored from 0 to 3, with a total score ranging from 0 to 84. Higher scores indicate more severe depressive symptoms.

The GAD-7 is a widely used self-report instrument for assessing the frequency and severity of anxiety symptoms over the past 2 weeks (23). It consists of 7 items, each scored from 0 to 3, with a total score ranging from 0 to 21. Higher scores indicate more severe anxiety symptoms.

The DARS is a self-report instrument used to assess anhedonia across different reward-related domains (24). It consists of 17 items covering four domains: hobbies, food and drink, social activities, and sensory experiences. Each item is scored from 0 to 4, yielding a total score ranging from 0 to 68. Higher scores indicate greater capacity for pleasure and lower levels of anhedonia.

The CUDOS-M is a self-report instrument used to assess mixed features during depressive episodes and is based on the DSM-5 mixed-features specifier (25). It assesses mixed-feature symptoms over the past week and consists of 13 items, each scored from 0 to 4, with a total score ranging from 0 to 52. Higher scores indicate more prominent mixed features.

### 2.4 Speech data collection

Spontaneous speech was collected using a semi-structured interview task. This task required participants to organize their verbal responses in a relatively natural conversational setting and involved recall of emotional experiences, information retrieval, cognitive processing, and language organization, thereby allowing the collection of relatively natural, continuous, and information-rich speech samples. The interview consisted of eight prompts: (1) describing an event within the past month that made the participant feel happy; (2) describing an event within the past month that made the participant feel angry; (3) describing a person or situation that made the participant feel afraid; (4) describing something that gave the participant a sense of hope; (5) describing an experience that caused substantial emotional hurt; (6) describing a memorable event from childhood; (7) describing the most important person in the participant’s life; and (8) describing how the participant viewed themselves as a person. The prompts covered emotional experiences, autobiographical memories, interpersonal relationships, and self-evaluation. None of these eight primary prompts directly asked about suicidal ideation, suicide plans, or suicidal behavior. A standardized interview guide was developed for each topic to define the core questioning points. Within this framework, participants were allowed to respond freely without strict constraints on the content or form of their responses.

Speech data were collected in a separate and quiet room to minimize external noise and environmental interference. All recordings were obtained using the same recording device, and the distance between the recording device and both the participant and interviewer was maintained at approximately 0.5 m. The recording device remained on throughout the interview to ensure the continuity and completeness of the speech data.

Participants who completed the 4-week follow-up underwent the same speech collection procedure as at baseline. The follow-up speech data were used to evaluate the performance of the baseline-trained models at a later assessment time point under the same speech task condition.

### 2.5 Speech data preprocessing

After speech data collection, the raw audio files were first uniformly numbered and organized, and the recordings were transcribed into structured JSON files containing text content and timestamp information. The transcription results were then manually checked by trained researchers, with a focus on correcting word recognition errors, omissions, sentence segmentation errors, and speaker identification errors, to improve the accuracy of subsequent textual analyses.

After manual correction, participants’ speech segments from the semi-structured interview task were extracted from the original audio files according to the corrected timestamp information. Interviewer speech and task-irrelevant content were removed. The segmented participant audio files were used for acoustic feature extraction, and the corrected participant response texts were exported as TXT files for traditional textual feature extraction and deep semantic feature extraction. To avoid potential influence from interviewer speech or text, all subsequent features were extracted only from participants’ responses.

### 2.6 Feature extraction

Speech-related features were extracted from the speech data collected during the semi-structured interview task. According to feature source and representation form, the extracted features were categorized into three types: acoustic features, traditional textual features, and deep semantic features. Speech data collected at the 4-week follow-up were processed using the same preprocessing and feature extraction procedures as those used at baseline to ensure feature consistency across time points.

Acoustic features were extracted from the segmented participant audio files using Librosa in Python and Praat (26, 27). For each participant, all retained speech segments from the semi-structured interview were first concatenated in chronological order to form a single participant-level waveform. Acoustic measures varying over time or frames were then summarized across this waveform to obtain fixed-length participant-level features. Specifically, root-mean-square energy, zero-crossing rate, and spectral centroid were represented by their mean values, whereas MFCCs, spectral flatness, spectral flux, fundamental frequency, and loudness were summarized using their mean and standard deviation. Sharpness was represented as a single participant-level value. Jitter, shimmer, harmonic-to-noise ratio, and formant frequencies were derived from the concatenated waveform using Praat. These procedures yielded 62 candidate acoustic features for each participant.

Traditional textual features were extracted from the manually corrected transcripts of participants’ responses. First, the Language Technology Platform (LTP) developed by Harbin Institute of Technology was used for Chinese word segmentation, part-of-speech tagging, and dependency parsing (28). Based on these analyses, lexical features, part-of-speech features, core-word features, and dependency-syntactic features were extracted to characterize participants’ lexical use, language organization, and syntactic structure during spontaneous speech. Emotion-related textual features were extracted using the Chinese Affective Lexicon Ontology (29). Words in participants’ transcripts were matched with the emotion lexicon to calculate the frequency, proportion, and emotional intensity indicators of different emotion categories, thereby reflecting the emotional content and affective tendency of participants’ language. The final traditional textual feature set consisted of 82 candidate features, including lexical, part-of-speech, syntactic, and emotion-word features.

Deep semantic features were extracted from the manually corrected transcripts of participants’ responses using the pretrained bert-base-chinese model as a fixed feature extractor without task-specific fine-tuning. For each participant, the interview transcript was tokenized and divided into consecutive chunks of up to 510 tokens, with [CLS] and [SEP] tokens added to each chunk. Each chunk was passed through BERT, and the 768-dimensional [CLS] representation from the final hidden layer was extracted. When multiple chunks were generated for a participant, the chunk-level [CLS] representations were averaged to obtain a single 768-dimensional participant-level semantic vector, which was used as the deep semantic input for model development.

### 2.7 Model development and internal validation

Suicide risk identification models were developed using the baseline sample, with the high suicide risk group defined as the positive class. To ensure comparability across models based on different feature sources, all models were developed using the random forest algorithm. Random forest improves model stability by integrating multiple decision trees and can handle high-dimensional features and nonlinear relationships, making it suitable for the multi-source feature data and binary classification task in this study (30). First, separate models were constructed using acoustic features, traditional textual features, and deep semantic features. A clinical-feature model was also constructed as a reference. The clinical-feature model included four prespecified clinical variables: the IDS-SR total score with the suicide-related item removed, GAD-7 total score, DARS total score, and CUDOS-M total score. To evaluate the incremental value of clinical symptom information beyond textual features, two additional models were developed: one combining traditional textual features with the IDS-SR score and another combining traditional textual features with all four prespecified clinical symptom measures. To directly assess the incremental contribution of traditional textual features beyond depressive symptom severity, an additional IDS-SR-only model was evaluated using the IDS-SR score excluding the suicide-related item.

Model development and internal validation were performed using stratified nested cross-validation. The outer loop used fivefold cross-validation to evaluate model performance, whereas the inner loop used threefold cross-validation for feature selection, dimensionality reduction, hyperparameter optimization, and classification threshold determination. All feature selection, dimensionality reduction, hyperparameter optimization, and threshold selection procedures were performed only within the training data, and the procedures determined in the training data were then applied to the corresponding test fold to avoid data leakage and overoptimistic performance estimates.

Because the IDS-SR-only and clinical-feature models included only one and four variables, respectively, all variables were retained and no feature selection was performed. The acoustic, traditional textual, traditional textual plus IDS-SR, and traditional textual plus all-clinical feature sets contained 62, 82, 83, and 86 candidate features, respectively. For these models, feature selection was performed within the cross-validation pipeline. Correlated features were first filtered using a Spearman correlation threshold of |ρ| > 0.80, followed by random-forest-importance-based Top-K selection, with K determined within the inner cross-validation loop. Across the five outer folds, the selected K values ranged from 15 to 35 for the acoustic-feature model, from 20 to 35 for the traditional textual-feature model, from 20 to 35 for the traditional textual plus IDS-SR model, and from 15 to 35 for the traditional textual plus all-clinical model. For the deep semantic model, the 768-dimensional participant-level BERT vectors were standardized and then reduced to 50 principal components using PCA before random forest modeling. Both standardization and PCA were implemented within the nested cross-validation pipeline, such that the transformations were fitted only on the corresponding training data and then applied to the corresponding held-out data.

### 2.8 Follow-up validation using 4-week follow-up data

After model development and internal validation using the baseline sample, the area under the curve (AUC) from internal validation was used as the primary criterion for model selection, together with the availability of corresponding features at follow-up. Accordingly, the RF model based on traditional textual features and the RF model combining traditional textual features with the IDS-SR total score were selected for follow-up validation. Because DARS and CUDOS-M were not repeatedly collected at follow-up, the combined model including all clinical features was not evaluated at follow-up. Among participants who completed the 4-week follow-up, those with a HAMD-17 total score of ≥14 at the follow-up visit were included in the follow-up validation.

The models used for follow-up validation were determined entirely based on the baseline sample. Specifically, features selected in at least four of the five baseline cross-validation folds were defined as stable features and used as the final feature set. Based on these features, RF models were retrained using the entire baseline sample, and the final classification threshold was determined using the same threshold selection strategy as that used at baseline. The resulting models and classification thresholds were then fixed and directly applied to the corresponding features obtained at the 4-week visit. Model performance was evaluated against suicide-risk groups redefined at the same visit. No data collected at the 4-week follow-up were used for feature selection, model training, hyperparameter optimization, or threshold determination. Importantly, this follow-up analysis did not assess prospective prediction of future suicide risk from baseline speech, as both the model inputs and suicide-risk labels used for follow-up evaluation were obtained at the 4-week visit.

### 2.9 Statistical analysis

Continuous variables were summarized as mean ± standard deviation or median with interquartile range, depending on their distribution. Categorical variables were summarized as frequencies and percentages. To compare baseline characteristics between the high and low suicide risk groups, continuous variables were analyzed using the independent-samples t test or the Mann–Whitney U test, as appropriate, and categorical variables were analyzed using the chi-square test or Fisher’s exact test. To assess potential attrition bias, baseline demographic and clinical characteristics were also compared between participants who completed the 4-week follow-up and those who did not, using the same statistical procedures. All statistical tests were two-sided, and P < 0.05 was considered statistically significant; for the pairwise AUC comparisons, statistical significance was based on Holm-adjusted P values.

In both internal validation and follow-up validation, model performance was evaluated using accuracy, sensitivity, specificity, F1 score, and the area under the receiver operating characteristic curve (AUC).

For both internal and follow-up validation, 95% confidence intervals (CIs) for AUC were estimated using nonparametric bootstrap resampling with 2,000 resamples. Internal-validation analyses were based on participant-level out-of-fold predicted probabilities from the outer cross-validation folds, whereas follow-up analyses used predictions generated by the fixed baseline-trained models on the 4-week data. Differences in AUC between selected competing baseline models were assessed using paired bootstrap resampling with 2,000 resamples, with 95% CIs and nominal two-sided P values derived from the paired bootstrap distributions. These pairwise AUC comparisons were targeted, non-exhaustive comparisons selected to address the study questions, but they were not formally prespecified as confirmatory hypotheses. To account for multiple comparisons across the pairwise AUC analyses, P values were adjusted using the Holm method. For internal validation, precision–recall curves and average precision (AP) were used to complement ROC-based evaluation; calibration was examined using 10-bin quantile-based calibration plots, and overall probabilistic prediction error was summarized using Brier scores. To further characterize the variability in model performance across the outer cross-validation folds, AUCs were calculated separately for each of the five outer test folds, and the mean, standard deviation, and range of the fold-specific AUCs were summarized.

To assess the sensitivity of the findings to the operational definition of suicide risk, a sensitivity analysis was conducted using an alternative outcome definition in which high suicide risk was defined solely by a BSI-CV total score of >10, without applying the HAMD-17 suicide-item screening step. The same model-development and nested cross-validation procedures used in the primary analysis were repeated under this alternative outcome definition.

## 3 Results

### 3.1 Baseline characteristics

A total of 248 patients with MDD were included at baseline, including 92 patients in the high suicide risk group and 156 patients in the low suicide risk group. Significant differences between the two groups were observed in age, IDS-SR total score, GAD-7 total score, and DARS total score, whereas no significant differences were found in the other demographic or clinical characteristics. There were also no statistically significant between-group differences in participant speech duration or transcript length (**Table 1**).

**Table 1:** Baseline characteristics of participants by suicide risk group.

Variable	High suicide risk group (n = 92)	Low suicide risk group (n = 156)	Statistic	*P* value
Male sex, n (%)	28 (30.4)	42 (26.9)	0.352	0.553
Age, years, median (IQR)	28.0 (11.0)	31.0 (12.0)	-2.071	0.038
Marital status, n (%)			7.653	0.054
Unmarried	70 (76.1)	96 (61.5)		
Married	18 (19.6)	53 (34.0)		
Divorced	2 (2.2)	6 (3.8)		
Widowed	2 (2.2)	1 (0.6)		
Education level, n (%)			3.281	0.350
Junior high school or below	5 (5.4)	4 (2.6)		
High school	16 (17.4)	25 (16.0)		
Bachelor’s degree	56 (60.9)	89 (57.1)		
Master’s degree or above	15 (16.3)	38 (24.4)		
Employment status, n (%)			4.085	0.130
Employed	54 (58.7)	100 (64.1)		
Student	18 (19.6)	37 (23.7)		
Retired or unemployed	20 (21.7)	19 (12.2)		
Per-capita monthly income, n (%)			1.735	0.629
<3000 CNY	5 (5.4)	6 (3.8)		
3000–4999 CNY	11 (12.0)	15 (9.6)		
5000–9999 CNY	30 (32.6)	44 (28.2)		
≥10000 CNY	46 (50.0)	91 (58.3)		
HAMD-17 total score, median (IQR)	20.0 (8.0)	19.0 (6.0)	-1.753	0.087
IDS-SR total score, median (IQR)	44.0 (23.0)	35.0 (14.0)	-4.725	<0.001
GAD-7 total score, median (IQR)	13.0 (8.0)	11.0 (7.0)	-3.031	0.003
DARS total score, median (IQR)	18.5 (19.0)	22.5 (22.0)	-2.858	0.008
CUDOS-M total score, median (IQR)	3.0 (6.0)	3.0 (6.0)	-0.942	0.306
Speech duration, min, median (IQR)	10.19 (7.01)	10.98 (7.04)	-1.139	0.255
Transcript length, segmented words, median (IQR)	1565.5 (1130.8)	1663.5 (1175.8)	-1.332	0.183

IQR, interquartile range. Continuous variables were compared using the Mann–Whitney U test, with standardized Z values reported as test statistics; categorical variables were compared using the chi-square test or Fisher’s exact test, as appropriate. Speech duration was calculated from the participant-only waveform, and transcript length as the number of LTP-segmented words in the corrected transcript.

### 3.2 Model performance in internal validation

The internal validation performance of the different random forest models is shown in **Table 2**. The IDS-SR-only model achieved an AUC of 0.625, while the clinical-feature model achieved an AUC of 0.574. Among the single-source speech-related models, the acoustic-feature, traditional textual-feature, and deep semantic-feature models achieved AUCs of 0.604, 0.687, and 0.641, respectively. The traditional textual-feature model achieved the highest AUC among the single-source speech-related models; however, the pairwise differences compared with the acoustic- and deep semantic-feature models were not statistically significant after Holm adjustment (**Table 3**).

**Table 2:** Internal validation performance of random forest models for suicide risk identification.

Feature set	Accuracy	Sensitivity	Specificity	F1 score	AUC (95% CI)	AP	Brier score
IDS-SR only	0.536	0.783	0.391	0.556	0.625 (0.551–0.700)	0.537	0.235
Clinical features	0.500	0.674	0.397	0.500	0.574 (0.500–0.652)	0.498	0.238
Acoustic features	0.544	0.717	0.442	0.539	0.604 (0.530–0.676)	0.436	0.243
Traditional textual features	0.597	0.717	0.526	0.569	0.687 (0.619–0.753)	0.537	0.212
Deep semantic features	0.589	0.717	0.513	0.564	0.641 (0.569–0.711)	0.511	0.220
Traditional textual features + IDS-SR total score	0.641	0.772	0.564	0.615	0.745 (0.682–0.811)	0.620	0.198
Traditional textual features + all clinical features	0.633	0.772	0.551	0.609	0.756 (0.694–0.819)	0.649	0.192

AP, average precision; CI, confidence interval. The Brier score represents the mean squared difference between predicted probabilities and observed binary outcomes, with lower values indicating better probabilistic prediction accuracy.

**Table 3:** Pairwise comparisons of AUCs between selected baseline models.

Comparison	ΔAUC	95% CI	*P* value	Holm-adjusted *P* value
Traditional textual vs Acoustic	0.083	−0.022–0.186	0.131	0.393
Traditional textual vs Deep semantic	0.046	−0.040–0.130	0.292	0.584
Traditional textual + IDS-SR vs Traditional textual	0.058	0.003–0.112	0.040	0.160
Traditional textual + IDS-SR vs IDS-SR only	0.120	0.046–0.198	0.003	0.018
Traditional textual + all clinical vs Traditional textual	0.069	0.016–0.126	0.010	0.050
Traditional textual + all clinical vs Traditional textual + IDS-SR	0.011	−0.020–0.042	0.495	0.584

ΔAUC was calculated as the AUC of the first model minus that of the second model. Nominal P values were derived from paired bootstrap comparisons with 2,000 resamples. Holm-adjusted P values account for multiple comparisons across the six pairwise AUC comparisons reported in this table.

Based on these findings, traditional textual features were further combined with clinical symptom measures. The model combining traditional textual features with the IDS-SR total score achieved an AUC of 0.745, compared with 0.687 for the traditional textual-feature model alone (ΔAUC = 0.058, 95% CI 0.003–0.112, P = 0.040; Holm-adjusted P = 0.160) and 0.625 for the IDS-SR-only model (ΔAUC = 0.120, 95% CI 0.046–0.198, P = 0.003; Holm-adjusted P = 0.018). The model combining traditional textual features with all clinical features achieved the highest AUC of 0.756 and was higher than the traditional textual-feature model by 0.069 (95% CI 0.016–0.126, P = 0.010; Holm-adjusted P = 0.050). The difference between the two combined models was 0.011 (95% CI −0.020–0.042, P = 0.495; Holm-adjusted P = 0.584). After Holm adjustment, only the difference between the model combining traditional textual features with the IDS-SR total score and the IDS-SR-only model remained statistically significant. Pairwise comparisons between selected baseline models are presented in **Table 3**. Precision–recall curves based on the out-of-fold predictions of the baseline models are shown in **Figure 1**, and calibration curves based on the out-of-fold predicted probabilities are shown in **Figure 2**. The models combining traditional textual features with the IDS-SR total score and with all clinical features showed the highest AP values (0.620 and 0.649, respectively) and the lowest Brier scores (0.198 and 0.192, respectively) among the baseline models.

**Figure 1:**
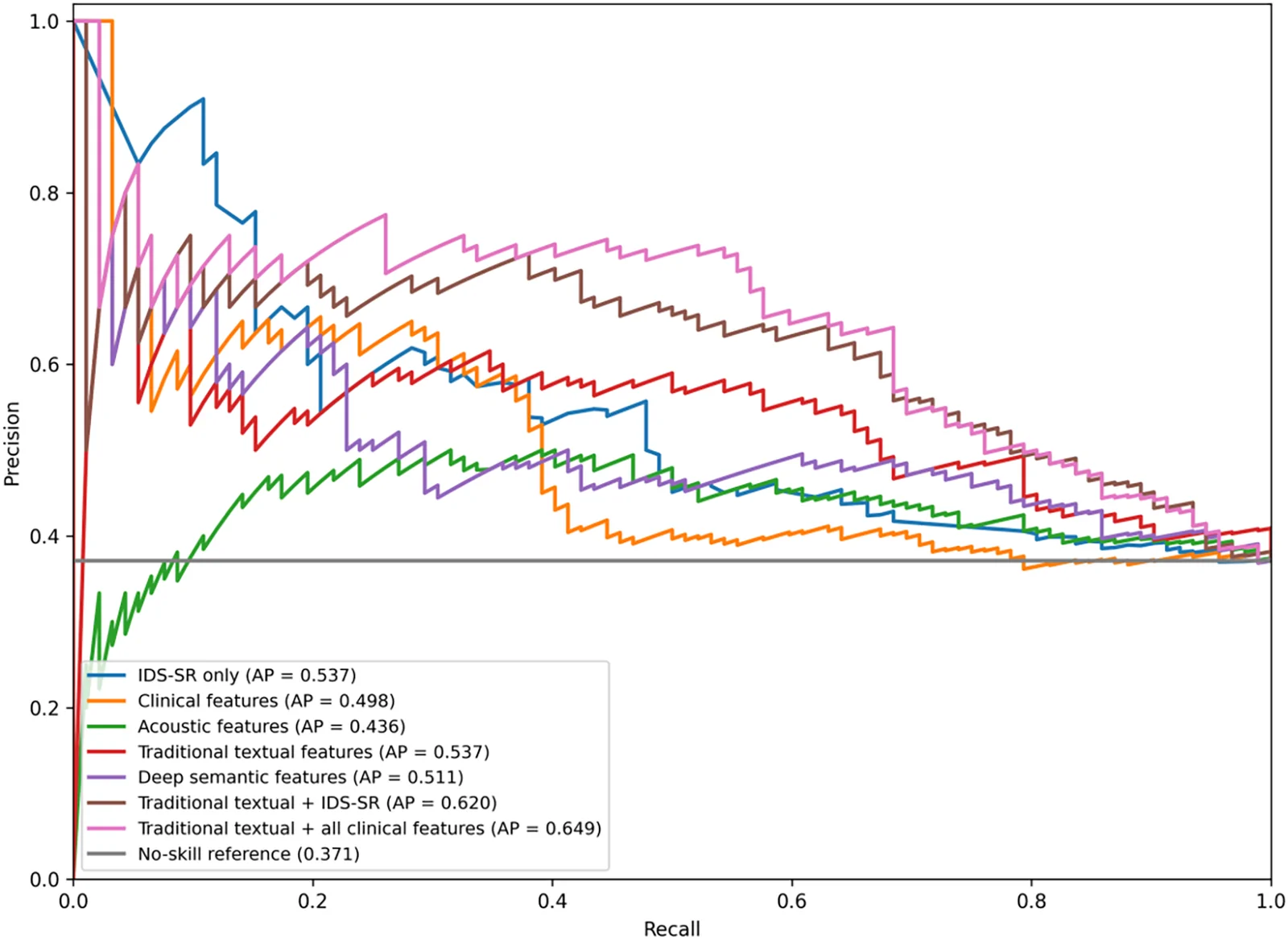
Precision–recall curves of the baseline models based on out-of-fold predictions.

**Figure 2:**
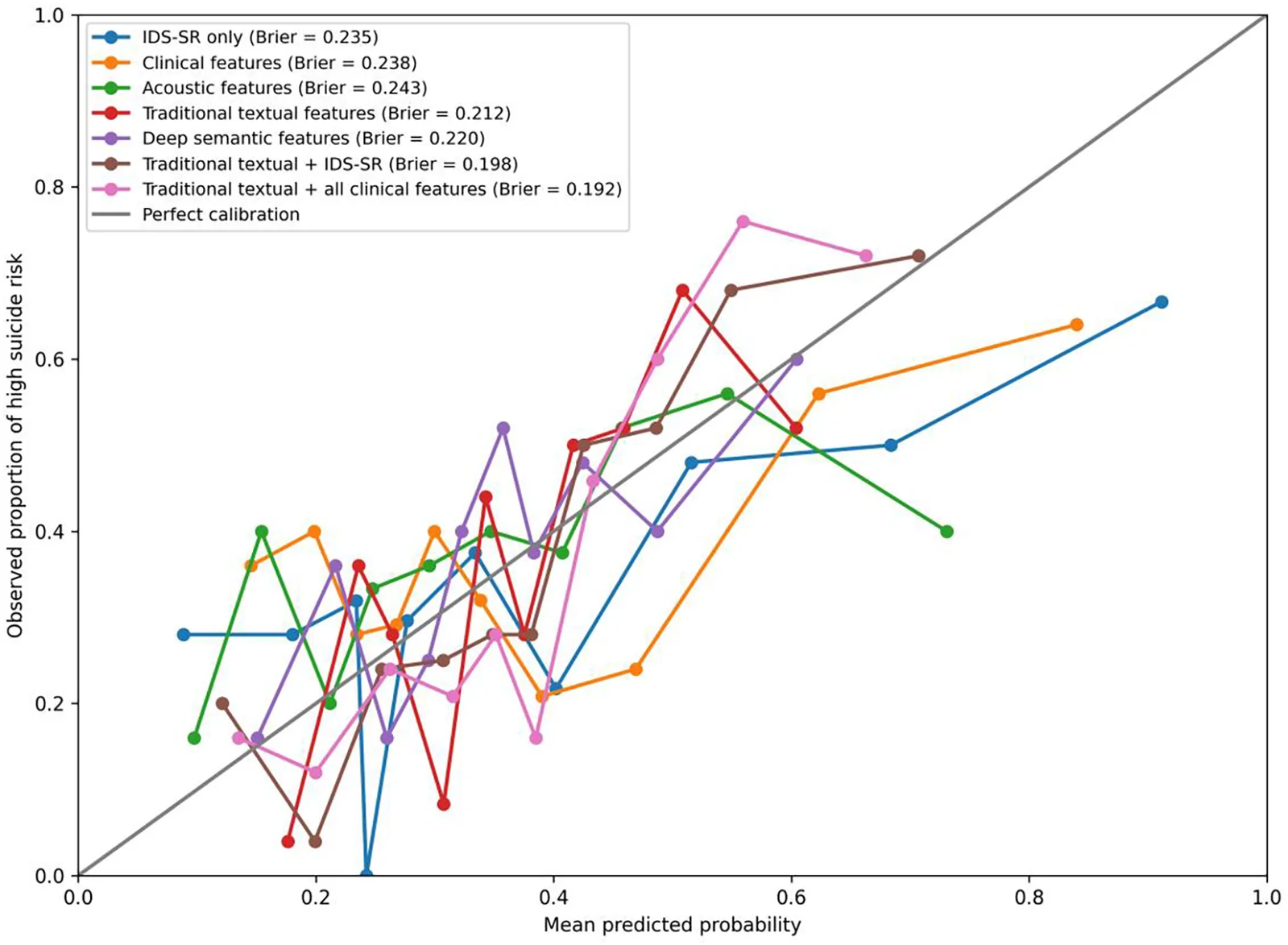
Calibration curves of the baseline models based on out-of-fold predicted probabilities.

Across the five outer test folds, the two models combining traditional textual features with clinical information showed mean AUCs of 0.753 (SD = 0.057) and 0.761 (SD = 0.057), respectively. Mean fold-specific AUCs for the remaining models ranged from 0.577 to 0.682, with SDs ranging from 0.033 to 0.068. Detailed fold-to-fold variability is summarized in **Supplementary Table 2**.

In the sensitivity analysis using an alternative operational definition of high suicide risk based solely on a BSI-CV total score of >10, model discrimination was generally lower than in the primary analysis. Nevertheless, the two models combining traditional textual features with clinical information remained the best-performing models, with both combined models achieving an AUC of 0.708. AUCs for the single-source models ranged from 0.580 to 0.620. Detailed comparisons with the primary analysis are presented in **Supplementary Table 3**.

### 3.3 Final model selection and stable selected features

Based on the internal validation results and the availability of corresponding features at follow-up, the traditional textual-feature RF model and the traditional textual-feature plus IDS-SR total score RF model were selected as the final models for subsequent follow-up validation. For each final model, features selected in at least four of the five baseline cross-validation folds were defined as stable selected features and used as the final feature set. Based on these final feature sets, the two models were retrained using the entire baseline sample and then applied to the 4-week follow-up data. The stable selected features of the final models are listed in **Table 4**.

**Table 4:** Stable selected features in the final random forest models.

Feature	Traditional textual-feature RF model	Traditional textual-feature + IDS-SR RF model
IDS-SR total score	—	5/5 (100%)
Adverb ratio	5/5 (100%)	4/5 (80%)
Idiom ratio	5/5 (100%)	5/5 (100%)
Interjection ratio	5/5 (100%)	5/5 (100%)
Colloquial particle ratio	5/5 (100%)	5/5 (100%)
Noun ratio	4/5 (80%)	5/5 (100%)
Verb ratio	4/5 (80%)	4/5 (80%)
Adjective ratio	4/5 (80%)	4/5 (80%)
Preposition ratio	5/5 (100%)	5/5 (100%)
Measure-word ratio	4/5 (80%)	—
Temporal-word ratio	5/5 (100%)	5/5 (100%)
Locative noun ratio	4/5 (80%)	—
Attributive relation ratio	5/5 (100%)	5/5 (100%)
Punctuation dependency relation ratio	5/5 (100%)	5/5 (100%)
Jianyu dependency relation ratio	5/5 (100%)	5/5 (100%)
Core-word verb ratio	5/5 (100%)	5/5 (100%)
Verb-complement relation ratio	4/5 (80%)	—
Adverbial modifier relation ratio	4/5 (80%)	—
Happy emotion-word ratio	5/5 (100%)	5/5 (100%)
Praising emotion-word ratio	5/5 (100%)	5/5 (100%)
Happy emotion-word count	5/5 (100%)	5/5 (100%)
Positive polarity emotion-word ratio	5/5 (100%)	—
Emotion-word ratio	4/5 (80%)	4/5 (80%)
Sadness-related emotional intensity density	4/5 (80%)	—

### 3.4 Follow-up validation performance of the final models

A total of 95 participants completed the 4-week in-person follow-up. Compared with participants who did not complete the 4-week follow-up, follow-up completers had significantly lower baseline GAD-7 scores, whereas no statistically significant differences were observed in the other examined baseline demographic or clinical characteristics, including baseline suicide-risk status (**Supplementary Table 1**). Among the 95 follow-up completers, 55 still had a HAMD-17 total score of ≥14 at follow-up and were therefore included in the follow-up validation analysis. Among these 55 participants, 17 were classified as high suicide risk and 38 as low suicide risk at follow-up. Between baseline and follow-up, 11 participants showed a change in suicide risk category, including 9 who changed from high to low suicide risk and 2 who changed from low to high suicide risk.

In the 4-week follow-up validation set, the traditional textual-feature RF model achieved an AUC of 0.720 (95% CI 0.553–0.872), whereas the RF model combining traditional textual features with the IDS-SR total score achieved an AUC of 0.774 (95% CI 0.607–0.918). The latter model therefore showed a numerically higher AUC in the follow-up sample (**Table 5**).

**Table 5:** Follow-up validation performance of the final random forest models using 4-week follow-up data.

Feature set	Accuracy	Sensitivity	Specificity	F1 score	AUC (95% CI)
Traditional textual features	0.600	0.764	0.526	0.542	0.720 (0.553–0.872)
Traditional textual features + IDS-SR total score	0.745	0.765	0.737	0.650	0.774 (0.607–0.918)

## 4 Discussion

This study provides a comparative clinical evaluation of different speech representations for suicide risk identification in patients with MDD within a unified and leakage-aware modeling framework. Using the same random forest classifier and nested cross-validation procedure across feature sets allowed differences in model performance to be interpreted primarily in relation to the information captured by the different feature sources rather than differences in modeling algorithms. Among the single-source speech representations, traditional textual features achieved the highest AUC, although the pairwise differences compared with the acoustic and deep semantic models were not statistically significant after Holm adjustment. Combining traditional textual features with clinical symptom information yielded higher AUCs than the traditional textual-feature model alone, although these differences did not remain statistically significant after Holm adjustment. Notably, the model combining traditional textual features with the IDS-SR score achieved a significantly higher AUC than the IDS-SR-only model after Holm adjustment, supporting incremental discriminative information from traditional textual features beyond depressive symptom severity. In addition, the better-performing text-related models retained discriminative ability when applied to newly collected speech at the 4-week follow-up, suggesting that their performance may be maintained at a later assessment time point; however, this finding should be interpreted cautiously given the relatively small and selected follow-up sample.

In this study, the numerically higher AUC observed for the traditional textual-feature model compared with the acoustic-feature model may be related to differences in the information captured by these two types of features. Traditional textual features characterize patterns in lexical use, syntactic organization, and emotional expression, which may capture psycholinguistic cues associated with suicide risk (12). Suicide risk is not only associated with depressed mood but may also involve psychological processes such as hopelessness, self-negation, cognitive rigidity, impaired interpersonal connectedness, and negative expectations for the future (31); these processes may be more readily expressed through patients’ natural narratives about their personal experiences, emotional states, and interpersonal relationships. Although the traditional textual model was based primarily on lexical, part-of-speech, syntactic, and emotion-word features rather than direct semantic representations, the potential influence of interview topics should still be considered. The lack of significant between-group differences in speech duration and transcript length also reduces concern that the observed discrimination was mainly driven by differences in speech quantity. In contrast, acoustic features primarily reflect how patients speak, including prosody, voice quality, and spectral characteristics, and their relationship with suicide risk may therefore be more indirect. Meanwhile, depression itself can lead to acoustic changes such as slowed speech, flattened prosody, and reduced speech activity (32), which may overlap with acoustic manifestations associated with high suicide risk and thereby attenuate acoustic differences between suicide risk groups. In addition, acoustic features are more susceptible to individual vocal habits, recording conditions, speaking distance, and fluctuations in speech intensity (33), which may further reduce their stability and discriminative performance in this study.

The deep semantic feature model did not show a performance advantage over the traditional textual feature model. This finding should be interpreted in light of the sample size and modeling conditions of the present study. Deep semantic vectors are usually high-dimensional, whereas the sample size in this study was relatively limited. Even after dimensionality reduction, model instability, overfitting, or loss of semantic information may still have occurred (34). In addition, although deep semantic features can capture the overall semantic information of text, their individual dimensions usually lack explicit linguistic meaning, which limits the interpretability of the model results. Under the current sample size and modeling framework, traditional textual features showed numerically higher discriminative performance, whereas the additional incremental value of deep semantic features needs to be further evaluated in larger samples and under more rigorous validation conditions.

Compared with previous studies using speech information to identify suicide risk in patients with MDD, the model performance in the present study was moderate (17–19). Previous studies differed in sample composition, speech tasks, input features, and validation strategies; some included heterogeneous samples such as healthy controls or patients with bipolar disorder, or used interview content directly related to suicide risk assessment as model input. In contrast, the present study focused on patients with MDD with HAMD-17 total scores of ≥14 and used nested cross-validation to reduce the risk of information leakage and overoptimistic performance estimates. Therefore, the observed performance may provide a more realistic estimate of the discriminative ability of speech-based approaches for suicide risk identification in patients with MDD.

This need for rigorous evaluation is also evident in recent clinical validation work on speech-based mental health assessment. Bauer et al. evaluated automated MDD assessment using naturalistic clinical interview recordings from patients in routine care and found that, although speech-based classification showed discriminative value, it did not outperform established depression scales (11). These findings highlight the challenges of translating speech-based approaches into routine clinical practice and underscore the importance of clinically relevant samples, reliable reference standards, and rigorous validation.

This study also found that combining clinical symptom information with traditional textual features was associated with higher discriminative performance than using traditional textual features alone. Specifically, both combined models achieved higher AUCs than the traditional textual-feature model alone, although these differences did not remain statistically significant after Holm adjustment. In contrast, the model combining traditional textual features with the IDS-SR score achieved a significantly higher AUC than the IDS-SR-only model after Holm adjustment, supporting incremental discriminative information from traditional textual features beyond depressive symptom severity. Taken together, these findings suggest that textual and clinical symptom information may provide complementary value. Clinical features can reflect multidimensional symptom states, including depression, anxiety, anhedonia, and mixed features (35), whereas textual features provide unstructured information from patients’ natural language expression and may capture individualized risk-related cues that are not fully covered by standardized scales (36). Notably, the AUCs of the model combining traditional textual features with all clinical features and the model combining traditional textual features with the IDS-SR total score were similar, and their difference was not statistically significant after Holm adjustment. This finding suggests that depressive symptom severity may account for a substantial proportion of the incremental clinical information associated with suicide-risk discrimination.

Given the dynamic nature of suicide risk in patients with MDD, internal validation using baseline data alone cannot determine how a model performs when applied at a later assessment time point (6). Previous studies using speech information to identify suicide risk have predominantly adopted cross-sectional designs and evaluated model discrimination at a single time point, leaving limited evidence regarding model performance when applied to newly collected follow-up speech. Therefore, we conducted a 4-week follow-up validation in which the models developed at baseline were fixed and applied to features obtained at the follow-up visit. Both the traditional textual-feature model and the model combining traditional textual features with the IDS-SR total score retained discriminative ability in the follow-up sample, with AUCs of 0.720 and 0.774, respectively. Importantly, this analysis did not assess prospective prediction of future suicide risk from baseline speech; rather, the fixed baseline-trained models were evaluated using features and suicide-risk labels obtained contemporaneously at the 4-week visit.

The clinical endpoint should also be interpreted in light of its psychometric definition. Suicide-risk status was defined using contemporaneous psychometric assessments rather than subsequent suicide attempts, suicide deaths, or other hard clinical outcomes. Accordingly, the reported model performance reflects discrimination of scale-defined suicide-risk status at the time of assessment rather than prediction of future suicidal behavior. Although suicidal ideation is clinically important, it is distinct from subsequent suicide attempts, and the factors associated with ideation, attempts, and suicide death may not fully overlap (35). In addition, scale-derived labels remain dependent on patients’ disclosure and the assessment context, despite the use of both clinician-rated and self-reported information. To examine the extent to which the modeling results depended on the operational definition of suicide risk, we conducted a sensitivity analysis using a BSI-CV total score of >10 alone. Although AUCs were generally lower under this alternative definition, the models combining traditional textual features with clinical information remained the best-performing models, suggesting that the relative advantage of combining textual and clinical information was preserved. Nevertheless, the findings should be interpreted in the context of a contemporaneous, scale-defined suicide-risk outcome.

From a clinical translation perspective, the findings of this study suggest that models based on speech-derived textual features may have potential value for assisting suicide risk identification in patients with MDD. Given the complexity and dynamic nature of suicide risk assessment, and considering that the current model performance is not yet sufficient to support its use as an independent risk determination or clinical decision-making tool, clinicians still need to integrate rating scale assessments, history of suicide attempts, current stressful life events, social support, and clinical interview information (37). Therefore, models based on speech-derived textual features may be more appropriately positioned as tools for risk alerting and clinical decision support, providing complementary information to help identify patients who require further assessment or closer follow-up within a multidimensional clinical information framework.

## 5 Strengths and limitations

The present study has several strengths. First, we restricted the study population to patients with MDD who had HAMD-17 total scores of ≥14, allowing the value of speech information for identifying suicide risk to be evaluated in a relatively homogeneous clinical sample and reducing heterogeneity caused by the inclusion of different populations. Second, we compared models based on different feature sources within a unified random forest modeling framework and used five-fold stratified nested cross-validation for model development and internal validation. All feature selection, dimensionality reduction, hyperparameter optimization, and threshold selection procedures were strictly performed within the training data, and model performance was evaluated in independent test folds, thereby reducing the risk of data leakage and overoptimistic performance estimates. Finally, we used 4-week follow-up data to evaluate whether the baseline-trained text-related models retained discriminative performance when applied to newly collected speech at a later assessment time point.

This study has several limitations. First, this was a single-center study with a relatively limited sample size, which may affect model stability and the generalizability of the findings. Second, given the sample size and the aim of maintaining model stability and comparability across feature sources, the present study used a unified random forest framework and did not evaluate more recent self-supervised speech representations or end-to-end multimodal architectures. Future studies with larger samples should examine whether these approaches provide incremental value beyond the acoustic, handcrafted linguistic, and BERT-derived textual representations evaluated in the present study. Third, although 4-week follow-up data were used to evaluate the models at a later assessment time point, the follow-up sample was derived from the same center and study cohort and therefore cannot replace rigorous external validation in independent populations. Moreover, follow-up completers had lower baseline GAD-7 scores than non-completers, whereas no statistically significant differences were observed in the other examined baseline characteristics. Nonetheless, potential attrition-related selection bias cannot be excluded. Fourth, the suicide-risk outcome was defined using contemporaneous psychometric assessments rather than subsequent suicide attempts or other hard clinical outcomes. Although clinician-rated and self-reported measures were combined, scale-derived labels remain subject to reporting and measurement limitations. Therefore, the reported model performance should be interpreted as discrimination of scale-defined suicide-risk status rather than prediction of future suicidal behavior. Fifth, the textual models were developed using manually corrected transcripts. Although speech collection itself is low-burden for participants, model performance may differ when transcripts are generated using a fully automated speech-recognition workflow, and the need for manual correction introduces additional implementation burden. Future studies should evaluate fully automated transcription and textual feature extraction under real-world clinical conditions.

Further research should optimize speech representations and modeling approaches using larger datasets and evaluate model performance in independent multicenter cohorts. Prospective studies with longer follow-up periods are also needed to determine the clinical utility and generalizability of these approaches. Future work may additionally integrate speech-derived information with other behavioral or physiological indicators to improve suicide risk identification and assess its potential role as an auxiliary clinical tool.

## 6 Conclusion

This study showed that, among patients with MDD and HAMD-17 total scores of ≥14, traditional textual features extracted from semi-structured interviews achieved the highest AUC among the single-source speech-feature models. Models combining traditional textual features with either the IDS-SR total score or all clinical features achieved higher AUCs than the traditional textual-feature model alone. The model combining traditional textual features with the IDS-SR score also achieved a higher AUC than the IDS-SR-only model, supporting the incremental value of traditional textual features beyond depressive symptom severity for suicide-risk identification in patients with MDD. Together, these findings suggest that interview-derived textual information and structured clinical symptom measures may provide complementary information for suicide risk identification. When the better-performing text-related models were applied to newly collected speech at the 4-week follow-up, they retained discriminative ability at the later assessment time point. Overall, these findings support the value of rigorously evaluating complementary speech-derived and clinical information for suicide risk identification in clinically defined psychiatric populations. Further validation in larger, independent multicenter cohorts is required to establish the generalizability and clinical utility of these approaches before clinical implementation.

## Data Availability

The datasets generated and analyzed during the current study are not publicly available due to ethical restrictions and participant privacy protection. De-identified data may be available from the corresponding author upon reasonable request and approval from the relevant ethics committee.
