# Supplementary material for: Speech-based identification of suicide risk in patients with MDD: model development and 4-week follow-up validation
Source: Front Psychiatry. 2026 Oct 13;17:1944154. doi: 10.3389/fpsyt.2026.1944154 (PMC13710235; doi:10.3389/fpsyt.2026.1944154)
Supplement: Supplementary file 1 [file Table1.docx]

Supplementary Table S1. Baseline characteristics of participants who completed and did not complete the 4-week follow-up

| Baseline characteristic | Follow-up completers (n = 95) | Non-completers (n = 153) | *P* value | |
| --- | --- | --- | --- | --- |
| Male sex, n (%) | 25 (26.3) | 45 (29.4) | 0.598 | |
| Age, years, median (IQR) | 29.0 (12.0) | 29.0 (10.0) | 0.948 | |
| Marital status, n (%) |  |  | 0.292 | |
| Unmarried | 70 (73.7) | 96 (62.7) |  | |
| Married | 21 (22.1) | 50 (32.7) |  | |
| Divorced | 3 (3.2) | 5 (3.3) |  | |
| Widowed | 1 (1.1) | 2 (1.3) |  | |
| Education level, n (%) |  |  | 0.349 | |
| Junior high school or below | 3 (3.2) | 6 (3.9) |  | |
| High school | 18 (18.9) | 23 (15.0) |  | |
| Bachelor's degree | 59 (62.1) | 86 (56.2) |  |  |
| Master's degree or above | 15 (15.8) | 38 (24.8) |  |  |
| Employment status, n (%) |  |  | 0.091 |  |
| Employed | 54 (56.8) | 100 (65.4) |  |  |
| Student | 28 (29.5) | 27 (17.6) |  |  |
| Retired or unemployed | 13 (13.7) | 26 (17.0) |  |  |
| Per-capita monthly income, n (%) |  |  | 0.055 |  |
| <3000 CNY | 8 (8.4) | 3 (2.0) |  |  |
| 3000–4999 CNY | 8 (8.4) | 18 (11.8) |  |  |
| 5000–9999 CNY | 32 (33.7) | 42 (27.5) |  |  |
| ≥10000 CNY | 47 (49.5) | 90 (58.8) |  |  |
| HAMD-17 total score, median (IQR) | 20.0 (5.0) | 21.0 (7.0) | 0.258 |  |
| IDS-SR total score, median (IQR) | 36.0 (14.5) | 40.0 (20.0) | 0.080 |  |
| GAD-7 total score, median (IQR) | 10.0 (8.5) | 12.0 (8.0) | 0.026 |  |
| DARS total score, median (IQR) | 22.0 (16.0) | 20.0 (22.0) | 0.051 |  |
| CUDOS-M total score, median (IQR) | 3.0 (6.0) | 3.0 (6.0) | 0.973 |  |
| Baseline suicide risk group, n (%) |  |  | 0.544 |  |
| High suicide risk | 33 (34.7) | 59 (38.6) |  |  |
| Low suicide risk | 62 (65.3) | 94 (61.4) |  |  |

IQR, interquartile range. Continuous variables were compared using the Mann–Whitney U test, and categorical variables were compared using the chi-square test or Fisher’s exact test, as appropriate.
