# Supplementary material for: Speech-based identification of suicide risk in patients with MDD: model development and 4-week follow-up validation
Source: Front Psychiatry. 2026 Oct 13;17:1944154. doi: 10.3389/fpsyt.2026.1944154 (PMC13710235; doi:10.3389/fpsyt.2026.1944154)
Supplement: Supplementary file 2 [file Table2.docx]

Supplementary Table S2. Variability in model discrimination across the outer folds of nested cross-validation

| Feature set | Pooled OOF AUC | Mean fold AUC ± SD | Range |
| --- | --- | --- | --- |
| IDS-SR only | 0.625 | 0.632 ± 0.050 | 0.569–0.699 |
| Clinical features | 0.574 | 0.577 ± 0.044 | 0.526–0.634 |
| Acoustic features | 0.604 | 0.608 ± 0.056 | 0.528–0.674 |
| Traditional textual features | 0.687 | 0.682 ± 0.068 | 0.604–0.747 |
| Deep semantic features | 0.641 | 0.647 ± 0.033 | 0.600–0.691 |
| Traditional textual features + IDS-SR total score Traditional textual features + all clinical features | 0.745  0.756 | 0.753 ± 0.057  0.761 ± 0.057 | 0.686–0.822  0.708–0.835 |

Fold-specific AUCs were calculated from the held-out test set of each outer cross-validation fold. Mean and standard deviation (SD) summarize AUC variability across the five outer folds. The pooled out-of-fold (OOF) AUC was calculated from the combined participant-level predictions across all outer test folds.
