# Supplementary material for: Speech-based identification of suicide risk in patients with MDD: model development and 4-week follow-up validation
Source: Front Psychiatry. 2026 Oct 13;17:1944154. doi: 10.3389/fpsyt.2026.1944154 (PMC13710235; doi:10.3389/fpsyt.2026.1944154)
Supplement: Supplementary file 3 [file Table3.docx]

Supplementary Table S3. Comparison of model discrimination under the primary and alternative operational definitions of suicide risk

| Comparison | Primary definition, AUC | Alternative definition, AUC |
| --- | --- | --- |
| Clinical features | 0.574 | 0.617 |
| Acoustic features | 0.604 | 0.580 |
| Traditional textual features | 0.687 | 0.618 |
| Deep semantic features | 0.641 | 0.620 |
| Traditional textual features + IDS-SR total score Traditional textual features + all clinical features | 0.745  0.756 | 0.708  0.708 |

The primary definition classified participants as having high suicide risk when both the HAMD-17 suicide-item score was >1 and the BSI-CV total score was >10. In the sensitivity analysis, high suicide risk was defined solely as a BSI-CV total score of >10. All models were redeveloped using the same nested cross-validation procedure as in the primary analysis.
